# Changes in Mental Health Needs during COVID-19 in the Republic of Georgia: A Longitudinal Follow-up Study

**DOI:** 10.2174/17450179-v18-e2208100

**Published:** 2022-08-22

**Authors:** Nino Makhashvili, Ketevan Pilauri, Amy Mulick, Jana Darejan Javakhishvili, Lela Sturua, Daniela C. Fuhr, Bayard Roberts

**Affiliations:** 1 Mental Health Resource Centre, Ilia State University, Q. Cholokashvili Av. 3/5. E 122. Tbilisi, Georgia; 2 Ilia State University, Q. Cholokashvili Av. 3/5. E 122. Tbilisi, Georgia; 3 Department of Non-communicable Disease Epidemiology, London School of Hygiene and Tropical Medicine, Keppel Street, London, UK; 4 Institute of Addictions, Ilia State University, Q. Cholokashvili Av. 3/5. E 122. Tbilisi, Georgia; 5 National Center for Disease Control and Public Health of Georgia, 99, Kakhety highway, P. Shotadze Tbilisi Medical Academy; 51/2 Ketevan Dedofali Ave, Tbilisi, Georgia; 6 Department of Health Services Research and Policy, London School of Hygiene and Tropical Medicine, 15-17 Tavistock Place, London, UK

**Keywords:** Mental health, Republic of Georgia, COVID-19 concern, Stressors, Symptoms, Mental disorders

## Abstract

**Background:**

To examine changes in COVID-19 stressors and symptoms of mental disorders in the Republic of Georgia.

**Methods:**

A longitudinal design was used. Following on from our study of May-June 2020, this follow-up study in January-March 2021 was conducted at: (i)an individual level with the same respondents involved in the May-June 2020 study (repeat responders/cohort); and (ii) at a population-wide level, using non-probabilistic sampling. Questionnaire sections covered: (i)demographic, socio-economic characteristics; (ii)level of burden caused by COVID-19-related stressors/concern; and (iii)symptoms of anxiety(GAD-7), depression(PHQ-9), PTSD(ITQ), adjustment disorder(ADNM8). Descriptive and multivariable regression analyses were conducted.

**Results:**

Among population-level survey respondents(N=1195), the probability of reporting mental ill health symptoms increased in 2021 compared to 2020 for PTSD(OR1.82), depression(OR1.40), adjustment disorder(OR 1.80), and marginally for anxiety(OR1.17). For the individual repeat respondents(N=455), the probability increased for depression(OR1.88) and adjustment disorder(OR2.56). The perceived burden of pandemic concern worsened in 2021 compared to 2020 for almost all stressors, particularly around access to health care, infecting others, and conflict in the home. PTSD was associated with an increased concern score from 2020 to 2021.

**Conclusion:**

Our study highlights the need to strengthen response strategies to address the elevated mental health needs related to COVID-19 in Georgia. It recommends increasing accessibility of early interventions and the need to modernise mental health services to strengthen access to care. It also calls for monitoring patterns of mental health disorders for better understanding and responses to mental health needs in Georgia.

## 1 INTRODUCTION

COVID-19 and the necessary societal responses to control it have been shown to increase the burden of mental disorders. The physical distancing measures can cause social isolation and a breakdown of social networks [1-3] while the economic impacts might further exacerbate psychological distress [1, 2, 4-6]. Outcomes such as PTSD and adjustment disorders might also occur in people who have traumatic experiences related to COVID-19, including their own illness, deaths or illness of family or friends, or the inability to care for sick family members [7-10]. A disproportionate mental health effect from COVID-19 may also be seen in people who already have a mental disorder [5, 11-13]. There is a strong need to better understand how COVID-19 and related control measures influence mental health needs at the community and individual levels [2, 4, 12, 14].

The Republic of Georgia took early public health measures to limit the spread of COVID-19 from early 2020 onwards, and this helped to suppress the virus when case numbers were still manageable, but this suppression was less successful by late 2020, which experienced a rise in cases and the re-introduction of public health strict measures to limit the spread of COVID-19 (see Supplementary File 1 for further information on these measures) [15, 16]. The impact of COVID-19 on mental health may be profound as neuropsychiatric disorders already amounted to 22.8% of the pre-COVID-19 burden of disease in Georgia [17]. The mental health system of Georgia is currently under reform but may not be able to adequately respond to the already high mental health treatment gap, which may further worsen in the context of COVID-19 [18, 19]. In an earlier study, we documented high levels of mental health needs in Georgia associated with concern about COVID-19, along with coping strategies utilised to address the stressors [20]. This follow-up study builds on our earlier study and represents the second round of the longitudinally planned survey. The aim of this study is to examine changes in concern about COVID-19 from 2020 to 2021 and its association with symptoms of mental disorders in the Republic of Georgia. The specific objectives are to examine changes in: (i) the perceived burden of COVID-19 related stressors; (ii) the association of concerns about COVID-19 with mental health symptoms; and (iii) factors associated with these concerns.

## 2 MATERIALS AND METHODS

A longitudinal design was used. Following on from our previous internet-based cross-sectional study in May-June 2020 using non-probabilistic sampling of the general population in Georgia aged 18 years and above [20], this follow-up study was conducted with two levels and groups from January to March 2021: (i) at an individual level with same cohort respondents involved in the May-June 2020 study who had agreed to participate in this follow-up study (repeat responders); and (ii) at a population-wide level using non-probabilistic sampling of the general population in Georgia aged 18 years and above, using social and traditional media, key agencies, and investigator networks. Please see Supplementary File 2 for further details on the recruitment strategy.

The follow-up 2021 online survey questionnaire replicated that of the May-June 2020 online survey, with four sections included. First, background demographic and socio-economic characteristics (Table **1**). Second, questions on the level of the burden caused by COVID-19 related concerns (19 stressors) over the previous month (Table **2** for individual items), with response options ranging from ‘no concern at all’ (=1) to ‘strongly burdened’ (=4) resulting in a score range from 19 to 76. Third, symptoms of anxiety (GAD-7 anxiety score >9 [21]); depression (PHQ-9 depression score >9 [22]); adjustment disorder (ADNM8 -score >18.4 [23, 24]), and PTSD (ITQ [25]. Details on the instruments, scoring and their psychometric properties are reported in our previous study [20]. Missing data from the 2021 study are reported in Table **1**.

Ethics approval was provided by the National Centre for Disease Control and Public Health in Georgia.

### 2.1 Statistical Analysis

Descriptive data between the 2020 and 2021 studies (Table **1**) were compared using Chi-sq tests for the populations and exact McNemar tests for individual repeat responders. For the analysis of changes from 2020 to 2021 in perceived burden and mental health outcomes, multivariable regression analysis was used (Table **2**), and unique IDs for each respondent were generated so the regressions could identify multiple responses belonging to the same individual. In this way, the population and individual results were pooled and modelled together to increase the power to detect effects. The population results were generated using a logistic regression model with cluster robust standard errors, where the ‘cluster’ is each individual. The ‘population change’ in outcomes from 2020 to 2021 was adjusted for age, sex, education, living location, perceived household economic situation and previous diagnoses of mental disorders. For the analysis of individual repeat responders, results were from a mixed-effects logistic regression model, adjusted for the same variables but with an additional ‘random intercept’ parameter to account for the non-independence of outcomes in the same individuals. This changes the interpretation so that the adjusted effect is for ‘an individual’s change’ in outcomes from 2020 to 2021.

With the pooled data, we also estimated the proportion of the Georgian population having each COVID concern and symptoms of mental disorders (Table **2**) after correcting for differences between the whole population and our survey populations. These proportions were calculated using coefficients estimated in the ‘population’ model: they represent the probabilities of a randomly selected individual in Georgia having each outcome. We calibrated the probabilities so that the age, sex and living location represent their distribution in the Georgian population (37.728% age 18-39, 17.264% 40-49, 18.479% 50-59, 26.522% 60+; 53.85% female, 46.14% male; 29.85% Tbilisi, 27.35% other regional centres, 42.8% rural/village) based on Georgian census data [26], and assumed that all the other variables are distributed in the Georgian population the way they are in this sample (*i.e*., used the observed proportions in this calculation).

For the analysis of factors independently associated with changes in concern about COVID-19 from 2020 to 2021, multivariable regression modelling was used. The dependent variable was a greater burden of concern about COVID-19, and this was developed by totalling the scores from the 19 questions on the level of burden of stressors. This was regressed on time interacted linear mixed models with random individual intercepts (to account for repeat responders) and a random slope (to allow the change to vary between individuals) and an unstructured covariance between the random effects. The model adjusted for factors that may be associated with changes in covid concern score (based on our first study [20]) of age, sex, living location, education, household size, household economic situation, health status, any NCDs, high risk occupations, working outside the home, forcibly displaced from armed conflict (internally displaced persons (IDPs)), at least moderate anxiety, at least moderate depression, PTSD, adjustment disorder, and previously diagnosed with a mental disorder. A backward stepwise procedure was used to select the final model. Interactions were removed from the variable if a p-value was above 0.10, and the model re-ran until all remaining interaction p-values were <0.10.

## 3 RESULTS

There were 1195 responders at the population level and 455 individual repeat responders in the 2021 survey (34% response rate from the cohort). The sample characteristics are provided in Table **1**, including comparisons with the May-June 2020 survey. As with the 2020 study, the respondents in 2021 were predominantly women, had completed higher education, and living in urban areas. However, respondents in the 2021 survey were more likely to be older, male, less educated, and living in more rural areas when compared to the 2020 study respondents.

At the survey population level, levels of PTSD symptoms were higher in 2021 (24%) than 2020 (12%) (P<0.0001). Similarly, depression symptoms were higher at the population level in 2021 (33%) compared to 2020 (30%) (P=0.031), and this was the case for adjustment disorder symptoms as well (40% in 2020, 52% in 2021, P<0.0001). Anxiety symptoms were stable between the two years. For the individual repeat respondents, only adjustment disorder was significantly higher in 2021 (49%) compared to 2021 (39%) (P<0.0001).

However, after adjusting for differences in key demographic and socio-economic variables in multivariable regression analysis (Table **2**), more change was evident. The probability among population-level survey respondents of reporting PTSD symptoms increased in 2021 compared to 2020 (OR 1.82 [95% CI 1.50, 2.20), along with depression (OR 1.40 [95% CI 1.21, 1.63]), and adjustment disorder (OR 1.80 [95% CI 1.57, 2.06]), while anxiety was only marginally higher (OR 1.17 [95% CI 1.00, 1.39]). For the individual repeat respondents, the probability increased in 2021 compared to 2020 for reporting depression (OR 1.88 [95% CI 1.34, 2.64]) and adjustment disorder (OR 2.56 [95% CI 2.00, 3.29]). The probability of anxiety and PTSD symptoms did not show a statistically significant change between 2020 and 2021, although both were estimated to be higher in 2021. Pooled across both years and adjusted for other model factors, there were more symptoms of anxiety, depression and adjustment disorder in women compared to men and in 18-39-year-olds compared with the older age groups, and more symptoms of PTSD in 18-39-year-olds (not shown).

After calibrating the probabilities of each disorder to the age, gender and living location distributions of the Georgian population, we estimated that the 2021 burden of anxiety, depression, PTSD, and adjustment disorder – 20%, 30%, 21% and 51%, respectively (Table **2**) – were slightly lower than the observed estimates of 24%, 33%, 24% and 52% (Table **1**). However, the increase in these symptoms from 2020 to 2021, as calculated from the re-calibrated change, is greater than the observed increase for all disorders except for PTSD, where the re-calibrated increase was slightly less than the observed data.

The burden of concern about COVID-19 worsened in 2021 compared to 2020 for both population survey respondents and individual repeat responders for the majority of the causes of concern (Table **2**). At the population-level, the probability of reporting a concern in 2021 compared to 2020 was highest for: ‘insufficient capacity of intensive care’ (OR 2.18 [95% CI 1.86, 2.57]); ‘access to routine health care’ (OR 1.78 [95% CI 1.46, 2.18]); ‘poor information from authorities about COVID-19 and related measures’ (OR 1.72 [95% CI 1.45, 2.03]); and ‘concern of infecting others with COVID-19’ (OR 1.61 [95% CI 1.49, 1.84]). Among individual repeat responders, the probability of increased concern in 2021 compared to 2020 was highest for concerns about: ‘conflict at home’ (4.43 [95% CI 1.60, 12.23]); ‘child care’ (OR 3.59 [95% CI 1.40, 9.15]); ‘insufficient capacity of intensive care’ (OR 3.44 [95% CI 2.49, 4.75]); concern of ‘infecting others with COVID-19’ (OR 2.20 [95% CI 1.73, 2.79]), and ‘poor information from authorities’ (OR 2.12 [95% CI 1.64, 2.75]). Only concern for ‘income loss’ significantly improved from 2020 to 2021, while some other specific concerns were not significantly different between 2020 and 2021 (home working, uncertainty about the duration and risk of the pandemic, restricted access to goods, violent assaults at home, and having no place of retreat). In general, pooled across both years and adjusted for other factors in the models, there were higher levels of concern for most measures in women compared to men and in 18-39-year-olds compared with the older age groups, though not all comparisons were statistically significant (not shown). In 2021, we estimated the greatest absolute population burden (by calibrated probabilities) to be ‘loved ones getting infected with COVID-19’ (69%), followed by ‘infecting others with COVID-19’ (62%) and ‘restricted contact with family and friends’ (49%).

Three factors were independently significantly associated with *changes* in concern about COVID-19 from 2020 to 2021. Being an IDP (coefficient of -2.99 [95% CI -4.97, -1.01]) and symptoms of anxiety (coefficient -3.00 [95% CI -4.50, -1.51]) were associated with a reduction in COVID-19 stressors score from 2020 to 2021. Conversely, having symptoms of PTSD was associated with increased COVID-19 concern from 2020 to 2021 (coefficient 1.88 [95% CI 0.08; 3.68]). The results of the full linear mixed model of factors associated with changes in COVID-19 concern between 2020 and 2021 are provided in Supplementary File **3**.

## 4 DISCUSSION

We found persistently high levels of symptoms of mental disorders, with the probability increasing in 2021 compared to 2020 for anxiety, depression, PTSD and adjustment disorder for the population-level study, and for depression and adjustment disorder for individual repeat responders/cohort members. The burden of mental disorders associated with COVID-19 is also evidenced in studies from other countries [3, 7, 8], and a systematic review reports a prevalence of anxiety and depression of 27% and 28%, respectively, during the COVID-19 pandemic [27]. In our study, the prevalence of anxiety and depression were respectively 24% and 33% in 2021. With regards to PTSD symptoms during COVID-19, a systematic review recorded levels varying from 7% to 53.8%, reporting moderate-to-severe psychological impact [28]. The prevalence of PTSD in our study was 24% in 2021, which was double the level in 2020 (12%).

It is noteworthy that respondents in our study repeatedly reported high levels of adjustment disorder symptoms (40% in 2020 and 52% in 2021). Research on adjustment disorder during COVID-19 is rather scarce despite researchers expressing concern about it due to COVID-19 [29]. We were able to identify only one study that measured the prevalence of adjustment disorder symptoms during the COVID 19 pandemic at multiple time points [30]. In that study (from Poland), 43% of respondents were reported as meeting the criteria for adjustment disorder in March 2020 and this increased to 52% in June 2020 [30]. These rates are similar to our findings in Georgia. However, a multi-country study in Europe recorded lower levels of adjustment disorder (18.2%) during the COVID-19 pandemic [31].

Several studies also indicated the changed trajectories of common mental disorders during the pandemic and related factors such as living in areas affected by lockdowns, struggling financially, having pre-existing conditions, or infection with COVID-19, while other studies documented a small increase in mental health symptoms soon after the outbreak of the COVID-19 pandemic that then decreased and was comparable to pre-pandemic levels by mid-2020. A recent study pointed to a significant decrease in depression and anxiety symptoms after the first shot of the vaccine. It seems that we are facing diverse and contrasting changes in mental health burden dependent on country-specific factors.

The increasing levels of mental health symptoms reported in our study are plausibly explained by the worsening COVID-19 infection rates in the country by the time of the second survey, along with much stricter measures that increased social isolation and the burden of stressors on the population and individual respondents (see Supplementary File 1). The findings from our study highlight the high and increasing burden of mental disorders in the country. The Ministry of Health in Georgia should recognise this in their annual mental healthcare program by providing promotional and preventive mental health services for the wider population in response to COVID-19 (*e.g*., through awareness raising and information sharing on common mental health conditions, increasing availability of psychoeducation for them, provision of self-help tips) and strengthening evidence-based treatment interventions (*e.g*., Cognitive-Behavioural Therapy (CBT), including trauma-focused CBT for PTSD). This also needs to be supported by a capacity strengthening program for both primary healthcare workers and mental health specialists for early identification and management of common mental health disorders. In addition, the mental healthcare packages offered by the central and local governments need to be strengthened, for example, by adding treatment for anxiety disorders and adjustment disorders which are currently not included (and so have to be paid for by patients). Ongoing monitoring is also required to detect changes in the patterns of mental disorders and ensure appropriate adjustments are made to the mental health care packages in Georgia.

The burden of concern about COVID-19 also worsened in 2021 compared to 2020 for the majority of the stressors, after adjusting for other factors. It was noticeable that the probability of reporting concern increased for health-service related factors such as concern about intensive care capacity, routine health care, and information on COVID-19 from authorities. This could be explained by significantly increased case-load in the country with increased mortality rates after the 2020 survey. Our findings correspond to the international evidence. For instance, a large-scale global cross-sectional study across 17 countries during the first and second wave of the COVID-19 pandemic reported almost a quarter (24%) of respondents had a high level of fear of COVID-19 [32]. Factors associated with poor mental health in studies on COVID-19 are diverse, but common ones reflect those in our study, such as concern about loved ones getting infected with COVID-19, infecting others with COVID-19, restricted social contact with family and friends, social isolation, and concern about insufficient capacity of hospital intensive care facilities [33]. In addition, there was a significant increase among individual repeat responders about conflict at home. After recalibration, it was estimated that around 10% of the population may be experiencing this. The increase implies a deterioration of family interactions due to lock-down measures, social isolation, schools and kindergartens closures, employment instability, etc. and corresponds to findings from other countries [34, 35]. The deterioration of the family dynamic should be noted both by professionals, policy-makers and social activists and relevant measures to address the increasing problem should be devised.

In our earlier study, we examined the factors associated with concern about COVID-19 [20], so in this study, we examined the factors associated with *changes* in concern about COVID-19 from 2020 and 2021. Having symptoms of anxiety was associated with a smaller-than-average increase in COVID-19 concern. Conversely, having symptoms of PTSD and being an IDP were associated with greater-than-average increases in the COVID-19 concern score in our follow-up study. The findings suggest that COVID-19 related concerns may be exacerbated among IDPs and other forcibly displaced persons who have faced many years of extreme stressors and a high ongoing burden of mental health disorders [36, 37]. Research on the topic of mental health and COVID-19 among IDPs is scarce [38], but our findings suggest that the Georgian government’s mental health policies and programs should consider the particular needs of IDPs and be adjusted accordingly. With regards to the association of symptoms of PTSD with increased COVID-19 concern score, while there has been debate over whether measures such as quarantine may meet traditional definitions of a traumatic event [39], COVID-19 and some control measures do appear to be associated with PTSD [40-42].

The international evidence highlights that frontline healthcare workers are among the most vulnerable groups affected by COVID-19 in terms of risk of developing mental health conditions such as depression, PTSD, anxiety and burnout syndrome [43-45]. However, in *post-hoc* analyses of our data, we found that healthcare workers had a significantly lower risk of depression, anxiety, PTSD and adjustment disorder than non-healthcare workers after adjusting for demographic differences between individuals. Despite this, we fear that the resilience shown in our study sample could rapidly deteriorate due to the length of the stress and unpredictable work-related burden caused by the pandemic, and urge healthcare management and policy-makers to adopt both preventive policies and treatment services [46].

This is one of the few follow-up studies to examine changes in the influence of concern about COVID-19 and its influence on mental health needs. Other follow-up studies have focused on specific groups, particularly those diagnosed with COVID-19 [47-49], rather than general populations. This study highlights the persistence of concern about COVID-19 and the ongoing mental health burden on the population and individual levels. The findings could be used to inform relevant policies, *i.e*., for addressing increased family violence problems, promoting mental wellbeing and developing prevention interventions, improving access to care for common mental disorders by mobilising resources at the primary healthcare level, and introducing capacity strengthening program for mental health specialists. This aligns with global calls for such activities to support mental health during the COVID-19 pandemic and re-balance mental health care towards community-oriented psychosocial perspectives, integrating mental health in plans for universal health coverage and addressing the needs of neglected populations to deal with disparities and inequality [50].

## 5 LIMITATIONS

The primary limitation is the use of non-probabilistic sampling due to the online survey methodology being used. This risks sample selection bias, for example, by excluding those without internet access who may have greater needs (such as those who have more severe mental disorders). Therefore, the findings cannot be interpreted to be prevalence data or as being nationally representative. However, basic descriptive analyses and explorations of potential associations can be appropriate in such surveys [51], and have been used in the majority of COVID-19 mental health surveys which have been done to date [52]. A key effect of the non-probabilistic sampling was the substantial over-representation of women in the study sample (with 88% of repeat responders and 72% of 2021 population-level responders being women), compared to 53% in census data from Georgia) [26], and this has been reported for online surveys previously [51]. We have responded to this by adjusting for gender in the regression analysis and recalibrating data when estimating the proportion of the Georgian population with the outcome. Other demographic data such as age distribution are more in line with Georgian census data, with the exception of an under-representation of those aged 60-69 (6% difference) and aged 70 and over (11% difference) [26], which is perhaps predictable given the online nature of the survey and likely lower use of the internet use among older people in Georgia. There was also a significant over-representation of respondents from Tbilisi compared with census data (39% difference) [26]. We adjusted our results for both of these factors too. We are unable to compare our findings on levels of mental health symptoms against nationally representative data as no such data have been released in Georgia and so a key recommendation is to share and collect new data from nationally representative random sampling surveys on mental disorders and their drivers in Georgia to demonstrate changes over time, as done in some other countries [53-55]. Second, self-reported mental health instruments are used for screening symptoms meeting criteria for mental health disorders only, rather than providing a mental health diagnosis. They have not been formally validated in Georgia, but they did show good internal reliability and construct validity with the study populations (which are also confirmed in previous studies which we have conducted in Georgia [36, 56]). Third, the limited number of respondents for some analyses limited the statistical power. Fourth, although we have adjusted for several factors that may be associated with changes in mental health outcomes, there may be unmeasured confounding affecting our regression results, and unmeasured or unknown distributions of factors that would affect our re-calibrated population probabilities if we had been able to account for them.

## CONCLUSION

Our survey in Georgia provides one of the few follow-up studies to examine changes in concern about COVID-19 stressors and mental health needs over time. It highlights persistently high and increasing levels of concern about COVID-19 and mental health symptoms. Government initiatives should consider tackling the most prevalent stressors related to COVID-19 to help strengthen their mental well-being of the Georgian population. Mental health infrastructure should be boosted to address the increased need for mental health services. This should include increased access to care for common mental health problems, providing preventive and early identification interventions, and using telephone and online services targeted and tailored to identified needs. The response should include mobilizing the primary healthcare level that is still divorced from mental health care in many post-Soviet countries, including Georgia. Public health researchers and clinicians also need to be mindful of COVID-19 and related responses exacerbating mental health disparities, and so vulnerable groups should be prioritised. Actions taken now may help prevent and mitigate the negative impact of COVID-19 on mental health in Georgia.

## Figures and Tables

**Table 1:** Sample Characteristics, by study type and period.

-	**Population-Level Responders ^i^**			**Individual Repeat Responders**		
-	**2020 Survey n (%)**	**2021 Survey** **n (%)**	**chi-sq *P***	**2020 Survey** **n (%)**	**2021 Survey** **n (%)**	McNemar* P
**Total Sample**	**2080 (100)**	**1195 (100)**		**455 (100)**	**455 (100)**	
**Age (years):**						
18-39	1176 (56.54)	499 (41.76)	<0.0001	251 (55.16)	247 (54.29)	
40-49	433 (20.82)	308 (25.77)		104 (22.86)	103 (22.64)	
50-59	322 (15.48)	201 (16.82)		70 (15.38)	70 (15.38)	
60-69	132 (6.35)	135 (11.30)		28 (6.15)	32 (7.03)	
70+	17 (0.82)	52 (4.35)		2 (0.44)	3 (0.66)	
**Gender:**						
Female	1801 (86.59)	860 (71.97)	<0.0001	400 (87.91)	402 (88.35)	
**Education:**						
Without education, primary only/ incomplete secondary	35 (1.68)	108 (9.04)	<0.0001	4 (0.88)	2 (0.44)	
Completed secondary education (including vocational)	113 (5.43)	191 (15.98)		17 (3.74)	19 (4.18)	
Not finished higher education	344 (16.54)	99 (8.28)		54 (11.87)	53 (11.65)	
Completed higher education	1588 (76.35)	797 (66.69)		380 (83.52)	381 (83.74)	
**Living location:**						
Tbilisi	1444 (69.42)	544 (45.52)	<0.0001	339 (74.51)	335 (73.63)	
Regional centre	463 (22.26)	272 (22.76)		89 (19.56)	91 (20.00)	
Village	173 (8.32)	379 (31.72)		27 (5.93)	29 (6.37)	
**Household economic situation:**						
Very Good	38 (1.83)	20 (1.67)	0.0002	135 (29.67)	110 (24.18)	
Good	369 (17.74)	168 (14.06)		127 (27.91)	98 (21.54)	
Average	1269 (61.01)	824 (68.95)		108 (23.74)	135 (29.67)	
Bad	311 (14.95)	148 (12.38)		83 (18.24)	111 (24.40)	
Very bad	77 (3.70)	24 (2.01)		2 (0.44)	1 (0.22)	
missing	16 (0.77)	11 (0.92)		135 (29.67)	110 (24.18)	
**COVID concern score ^ii^:**						
Quartile 1 (18-37)	646 (31.06)	259 (21.67)	<0.0001	135 (29.67)	110 (24.18)	<0.0001
Quartile 2 (38-44)	496 (23.85)	240 (20.08)		127 (27.91)	98 (21.54)	
Quartile 3 (45-52)	505 (24.28)	308 (25.77)		108 (23.74)	135 (29.67)	
Quartile 4 (53-76)	418 (20.10)	381 (31.88)		83 (18.24)	111 (24.40)	
missing	15 (0.72)	7 (0.59)		2 (0.44)	1 (0.22)	
**Symptoms of mental disorders ^iii^:**						
Anxiety	490 (23.56)	289 (24.18)	0.7084	94 (20.66)	96 (21.10)	0.9099
Depression	618 (29.71)	399 (33.39)	0.0307	134 (29.45)	146 (32.09)	0.2127
PTSD	247 (11.88)	283 (23.68)	<0.0001	51 (11.21)	51 (11.21)	1.0000
Adjustment disorder	822 (39.52)	626 (52.38)	<0.0001	177 (38.90)	222 (48.79)	<0.0001
**Previously diagnosed mental disorder:**						
No	1716 (82.50)	1059 (88.62)	<0.0001	386 (84.84)	378 (83.08)	0.8145
Yes	300 (14.42)	103 (8.62)		61 (13.41)	58 (12.75)	
missing	64 (3.08)	33 (2.76)		8 (1.76)	19 (4.18)	
**Current NCDs:**						
Diabetes	52 (2.50)	78 (6.53)	<0.0001	10 (2.20)	11 (2.42)	1.0000
Hypertension	169 (8.13)	146 (12.22)	0.0001	36 (7.91)	40 (8.79)	0.3877
Cardiovascular disease	65 (3.13)	60 (5.02)	0.0064	11 (2.42)	10 (2.20)	1.0000
Cancer	22 (1.06)	29 (2.43)	0.0015	6 (1.32)	4 (0.88)	0.6250
Respiratory illness	78 (3.75)	58 (4.85)	0.1275	19 (4.18)	27 (5.93)	0.1153

Notes: ^i^ The 2020 figure for population-level responders includes individual-level responders who then completed the 2021 survey (*i.e*., N=455).^ii^ Higher score means greater concern about COVID-19.^iii^ At least moderate level anxiety and depression (GAD-7 anxiety score >9; PHQ-9 depression score >9); ADNM 8 adjustment disorder score >18.4; see methods section for PTSD (ITQ) scoring.

**Table 2:** Changes from 2020-2021 in the perceived burden of concern and mental health outcomes (regression results).

-	N Survey Responses ^i^	N Individuals ^i^	Population Change 2020-21OR (95% CI)	Individual Change 2020-21OR (95% CI)	Estimated Proportion of Georgian Population with Outcome ^ii^	
2020	2021
**Mental disorders symptoms:** ^iii^						
At least moderate anxiety	3621	3162	**1.17 (1.00, 1.39)**	1.45 (0.79, 2.67)	0.1693	0.1950
At least moderate depression	3621	3162	**1.40 (1.21, 1.63)**	**1.88 (1.34, 2.64)**	0.2298	0.2980
Overall PTSD	3621	3162	**1.82 (1.50, 2.20)**	2.09 (0.86, 5.08)	0.1300	0.2134
Adjustment disorder	3621	3162	**1.80 (1.57, 2.06)**	**2.56 (2.00, 3.29)**	0.3635	0.5064
**Burden of concern:** ^iv^						
Infecting others with C19	3467	3036	**1.61 (1.40, 1.84)**	**2.20 (1.73, 2.79)**	0.5074	0.6234
Income loss	2986	2639	**0.82 (0.70, 0.97)**	**0.73 (0.56, 0.97)**	0.4485	0.4006
Becoming infected	3601	3150	**1.47 (1.26, 1.70)**	**1.88 (1.42, 2.50)**	0.3056	0.3921
Being at home	3416	2993	**1.22 (1.06, 1.41)**	**1.40 (1.11, 1.76)**	0.2907	0.3341
Restricted contact with family & friends	3576	3127	**1.36 (1.19, 1.55)**	**1.73 (1.37, 2.18)**	0.4097	0.4855
Loved ones get infected with C19	3601	3142	**1.25 (1.09, 1.44)**	**1.51 (1.18, 1.92)**	0.6456	0.6949
Insufficient capacity of intensive care	2860	2567	**2.18 (1.86, 2.57)**	**3.44 (2.49, 4.75)**	0.2778	0.4564
Home working	2371	2046	1.15 (0.92, 1.42)	1.55 (0.75, 3.20)	0.1246	0.1403
Restricted everyday activities	3565	3113	**1.22 (1.05, 1.41)**	**1.42 (1.11, 1.80)**	0.2214	0.2570
Childcare	2132	1940	**1.41 (1.14, 1.74)**	**3.59 (1.40, 9.15)**	0.1579	0.2088
Being socially isolated	3520	3075	**1.43 (1.24, 1.64)**	**1.87 (1.46, 2.38)**	0.2843	0.3622
Access to routine healthcare	3413	3013	**1.78 (1.46, 2.18)**	**1.95 (1.49, 2.54)**	0.1358	0.2187
Uncertainty on duration/risks of C19 pandemic	3600	3144	0.92 (0.80, 1.06)	0.90 (0.75, 1.09)	0.3930	0.3739
Poor information from authorities	3529	3087	**1.72 (1.45, 2.03)**	**2.12 (1.64, 2.75)**	0.1544	0.2386
Lack of food and essential goods	3459	3041	1.02 (0.82, 1.25)	1.05 (0.78, 1.40)	0.1372	0.1390
Violence at home	1800	1641	1.44 (0.92, 2.25)	1.77 (0.75, 4.16)	0.0613	0.0859
Restricted home space	3172	2800	**1.25 (1.05, 1.49)**	1.42 (0.76, 2.64)	0.1525	0.1839
No place of retreat	3087	2729	1.06 (0.89, 1.25)	1.10 (0.87, 1.39)	0.1685	0.1764
Conflict at home	2540	2259	**1.38 (1.08, 1.76)**	**4.43 (1.60, 12.23)**	0.1016	0.1351

Notes:^i^ Combined from 2020 and 2021 study populations.^ii^ Proportions estimated from the ‘population’ model with age, gender and living location recalibrated from the observed proportions in Table **1** to the demography of the Georgian population (see methods section).^iii^ At least moderate level anxiety and depression (GAD-7 anxiety score >9; PHQ-9 depression score >9); ADNM 8 adjustment disorder score >18.4; see methods section for PTSD (ITQ) scoring.^iv^ Respondents who answered ‘strongly burdened’ to individual items of COVID-19 concern.

## Data Availability

Data can be made available upon request to the corresponding author.

## LIST OF ABBREVIATIONS

COVID-19: Coronavirus
PTSD: Post-traumatic Stress Disorder
